# Rapid Evolution of Sperm Produces Diverse Centriole Structures that Reveal the Most Rudimentary Structure Needed for Function

**DOI:** 10.3390/cells7070067

**Published:** 2018-06-26

**Authors:** Tomer Avidor-Reiss

**Affiliations:** Department of Biological Sciences, University of Toledo, Toledo, OH 43607, USA; Tomer.AvidorReiss@utoledo.edu; Tel.: +1-419-530-1992

**Keywords:** centriole, centrosome, cilium, sperm, spermatogenesis, centrosome reduction, centriole remodeling, evolution

## Abstract

Centrioles are ancient subcellular protein-based organelles that maintain a conserved number and structure across many groups of eukaryotes. Centriole number (two per cells) is tightly regulated; each pre-existing centriole nucleates only one centriole as the cell prepares for division. The structure of centrioles is barrel-shaped, with a nine-fold symmetry of microtubules. This organization of microtubules is essential for the ancestral function of centriole–cilium nucleation. In animal cells, centrioles have gained an additional role: recruiting pericentriolar material (PCM) to form a centrosome. Therefore, it is striking that in animal spermatozoa, the centrioles have a remarkable diversity of structures, where some are so anomalous that they are referred to as atypical centrioles and are barely recognizable. The atypical centriole maintains the ability to form a centrosome and nucleate a new centriole, and therefore reveals the most rudimentary structure that is needed for centriole function. However, the atypical centriole appears to be incapable of forming a cilium. Here, we propose that the diversity in sperm centriole structure is due to rapid evolution in the shape of the spermatozoa head and neck. The enhanced diversity may be driven by a combination of direct selection for novel centriole functions and pleiotropy, which eliminates centriole properties that are dispensable in the spermatozoa function.

## 1. Spermatozoa Are a Highly Specialized Cell Type

**Figure 1:**
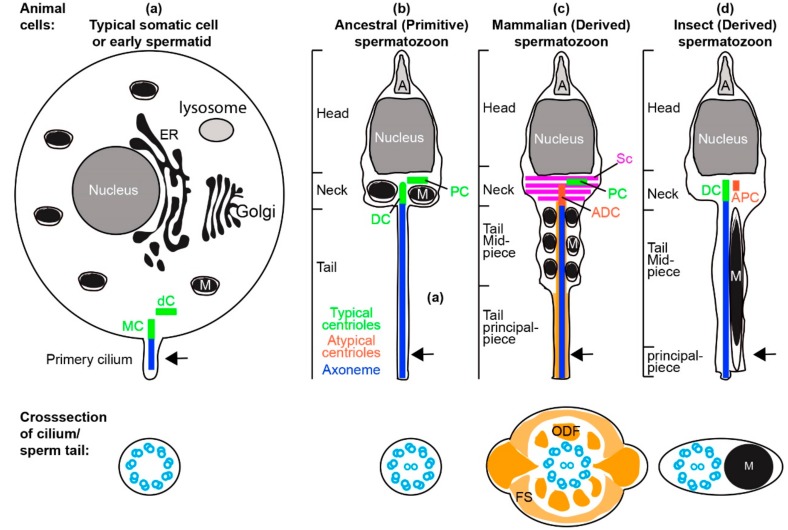
Centrioles in somatic cell and spermatozoa. (**a**) A representative undifferentiated somatic cell (e.g., stem cell in G1 phase of the cell cycle) or early spermatid with all organelles, and two typical centrioles: the mother centriole (MC) and daughter centriole (dC), and a short non-motile primary cilium. The primary cilium contains an axoneme made of nine doublets of microtubules. (**b**) Model of the ancestral “primitive” spermatozoa made of head, neck, and tail. The neck contains mitochondria (M) and two typical centrioles: the distal centriole (DC) and proximal centriole (PC). The Golgi forms the acrosome (A), the nucleus reshapes, and the endoplasmic reticulum (ER) is dramatically modified to a remnant. The sperm tail contains an axoneme made of nine doublets of microtubules with two central singlet microtubules. (**c**) Model of the mammalian spermatozoa made of head, neck, and tail that are divided into a midpiece and principle piece. The neck contains one typical proximal centriole and one atypical distal centriole. Additionally, the neck contains accessory structures that exhibit bilateral symmetry (e.g., striated column, SC). The mitochondria are found with the cytoplasmic axoneme in the midpiece [13]. The sperm tail contains the axoneme made of nine doublets of microtubules with two central singlet microtubules and accessory structures (outer dense fiber, ODF, and fibrous sheath, FS) that exhibit bilateral symmetry. (**d**) Model of the insect spermatozoa made of head, neck, and tail that is mostly a midpiece and with a tiny principle piece. The neck contains one typical distal centriole and one atypical proximal centriole. The sperm tail contains the axoneme made of nine doublets of microtubules with two central singlet microtubules. Asymmetrically located mitochondria are found along one side of the cytoplasmic axoneme along most of the sperm tail; this section is homologues to the midpiece in mammalian sperm [14]. Arrow in the animal cells indicated the plane of the cross-section in the cilium/sperm tail.

The spermatozoon is a very specialized cell type that has mainly one function: to fertilize an egg. To fertilize, the spermatozoon has a unique set of requirements. It must reach and penetrate the egg to complement its DNA and cytoplasmic content and initiate functional cell divisions. Thus, the structure and function of the spermatozoon is restricted by these specific requirements. These requirements evolved early in animal evolution and the spermatozoon acquired a simple structure and composition (Figure 1). The spermatozoon has a streamlined shape with three major components: head, neck, and tail. The head has a specialized nucleus with half of the genetic material of the offspring and a sperm-specific structure called the acrosome, which assists with sperm penetration to the ovum. The sperm neck contains centrioles that form the cilium (also known as the flagellum) of the sperm, and after fertilization form the major microtubule-organizing center of the zygote. The sperm neck or tail (depending on the species) also contains the mitochondria that are used as an energy source. The sperm tail, which is a cilium, has an axoneme as the motor that propels the sperm cell. In addition, the spermatozoon contains an egg activation factor PLCzeta [1], and RNA that functions in the zygote [2]. Taken together, these characteristics demonstrate that a spermatozoon exhibits a minimal set of highly specialized requirements.

In contrast to the specialized set of spermatozoon requirements, the spermatozoon is significantly less limited by other constraints that apply to the function of most other cell types. Most prominently, unlike somatic cells, the spermatozoon is not constrained by many ‘housekeeping’ functions, and it has minimal active gene transcription and reduced protein synthesis. As a result, the spermatozoon has transformed Golgi, little cytoplasm, residual ER, and no or inactive 80S cytoplasmic ribosomes (Figure 1) [3]. Therefore, it appears that during evolution, the relaxed constraints allowed anything that was not directly essential for fertilization to be minimalized or eliminated.

Another unique evolutionary feature of the spermatozoon is that it undergoes direct selection. Unlike other cells that contribute to the overall ability of an individual to secure a mate, spermatozoa from different individuals compete directly for the fertilization after mating. Thus, the spermatozoon demonstrates unique postcopulatory sexual selection such as sperm competition and cryptic female choice [4,5,6]. Sperm competition takes place when females have multiple mates and the sperm of the different males race to fertilize the eggs. Cryptic female choice takes place when a female selects the sperm of a particular male out of her mates to fertilize her egg [7]. This direct selection drives the spermatozoa to rapidly evolve.

Due to this direct selection, specialized requirements, and relaxed constraints, sperm cells are different from somatic cells. In addition, there are remarkable distinctions between the sperm cells of different animals. These distinctions can reflect differences in reproductive biology. For example, animals initially evolved in water and utilized external fertilization; their spermatozoon adapted to swim in the water. Later, some animal groups evolved to have internal fertilization, and their spermatozoon adapted to traversing the female reproductive tract and being stored for a long time. These changes resulted in a transition from an ancestral “primitive” short form of sperm consisting of a head, neck, and tail to a longer sperm consisting of a head, neck, midpiece, and principle piece (Figure 1) [8]. In parallel, the mitochondria, which normally function as an energy source of the cell, evolved a structural role in reducing the flexibility of the tail midpiece. Specialized structures (striated column, outer dense fiber, and fibrous sheath) evolved around the centriole and axoneme (Figure 1c).

**Figure 2:**
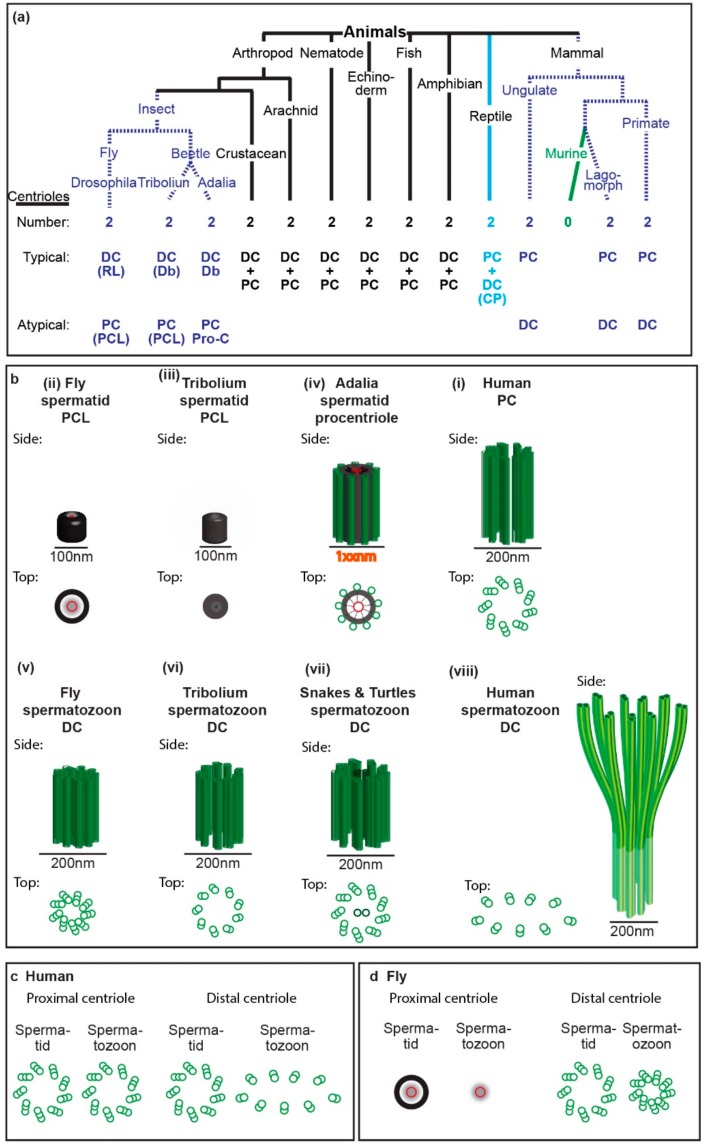
Spermatozoon typical and atypical centrioles have diverse structures in animals. (**a**) Phylogenetic tree with representative animal groups. Number of centrioles (first row), which centriole is typical and atypical (second and third rows, respectively), and type of centrioles as well as their modifications (in parenthesis) are indicated; RL, reduced diameter of the centriole lumen; Db, nine doublets of microtubules instead of triplets; PCL, proximal centriole-like structure as depicted in panel “b” or “d”; Pro-c, procentriole; CP, centriole with central pair. (**b**) Models of spermatid and spermatozoa proximal and distal centriole. The centrioles are depicted from side and top views and are to scale. (**c**,**d**) The structural differences between spermatid centrioles and spermatozoa centrioles in human (**c**) and fly (**d**).

**Figure 3:**
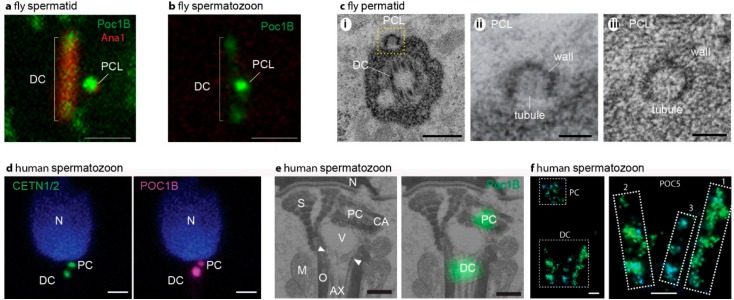
Sperm centrioles in flies (**a**–**c**) and mammals (**d**–**f**). (**a**) Fluorescent microscope picture of fly spermatid with DC and PCL labeled by the centriolar protein Poc1B and Ana1. (**b**) Fluorescent microscope picture of fly spermatozoon with Poc1B faintly labeling the DC and intensely labeling the PCL. In (**a**,**b**) the scale bar is 1 μm, Poc1B is genetically tagged by GFP, and Ana1 is genetically tagged by tdTomato. (**c**) Electron microscope picture of fly spermatozoon centrioles. (**i**) A section in a plane that has a cross-section of the DC and longitudinal section of the PCL (yellow box). (**ii**) Zoom in the PCL boxed in (**i**). (**iii**) A cross-section of the PCL depicting the wall and central tubule. In (**c**), the scale bar is 0.1 μm. (**d**) Fluorescent microscope picture of a human spermatozoon with CENTE1/2 or POC1B labeling the DC and PCL. The scale bar is 1 μm. CENTE1/2 and Poc1B are labeled by specific antibodies. (**e**) Correlative light and electron microscopy picture of human spermatozoon centrioles. On the left, electron microscopy section depicting POC1B labeling of the PC that is found near the nucleus, and the DC that is attached to the axoneme. Ax, axoneme; M, mitochondria; mp, tail midpiece; N, nucleus; ne, neck; O, outer dance fibers; S, striated columns; V; vault; CA; centriole adjunct. (**f**) Stochastic Optical Reconstruction Microscopy (STORM) super-resolution fluorescent microscopy picture of bovine spermatozoon centrioles. On the left, a picture depicting POC5 labeling of the PC and the DC. On the right, zooming in on the DC identifies two major rods (marked as “1” and “2”) and one minor rod (marked as “3”) labeled by POC5. In (**f**), the scale bar is 0.1 μm. Panels (**a**–**c**) are from Khire 2016 [15]. Panels (**d**–**f**) are from Fishman [9].

The ancestral “primitive” sperm had two structurally recognizable centrioles (typical centrioles) in its neck. However, multiple groups of animals evolved a sperm neck with one or no typical centrioles (Figure 1 and Figure 2a). Recent studies suggest that some of the species that were thought to have only one centriole actually have a second centriole with an atypical structure (atypical centriole) (Figure 3) [9,10,11,12]. In this manuscript, we will describe centriole evolution by focusing on sperm that has one typical centriole and one atypical centriole. These centriole types will be discussed in light of recent emerging data in two major groups of animals, insects, and mammals.

## 2. The Typical Centriole and the Role of the Centriole Microtubules

**Figure 4:**
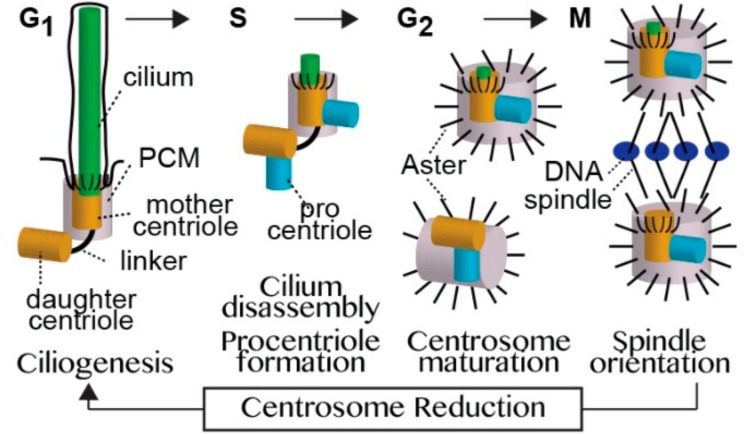
A model of the centriole cycle. Cells have two centrioles: the mother centriole and daughter centriole. The two centrioles have distinct roles in controlling cilium number, and in some cell types, in the outcome of asymmetric cell division. The mother centriole is the older of the two centrioles and is compositionally and structurally fully developed (mature). However, its function differs depending upon the present stage of the cell cycle. When a cell is in G1 phase, the mother centriole is enveloped by a thin layer of pericentriolar material (PCM) and assembles a cilium. In S phase, the cilium disassembles and the mother centriole forms a new centriole (the procentriole). In G2 phase, the mother centriole recruits the PCM, which nucleates and anchors microtubules to form an aster. In M phase, the mother centriole participates in the formation and localization of one of the mitotic spindle poles. After the completion of cell division, each centriole pair becomes a mother and a daughter centriole for one of the two daughter cells, and the mother centriole loses most of its PCM and astral microtubules through a process known as centrosome reduction. The daughter centriole is the younger of the two centrioles, and is compositionally, structurally, and functionally immature. When the cell is in S phase, the daughter centriole forms a procentriole. In G2 phase, the daughter separates from its mother, recruits PCM, forms an aster, and then forms the second centrosome. In M phase, the daughter centriole participates in the formation and localization of the second mitotic spindle pole, and after cell division becomes a mother centriole for one of the daughter cells.

In a typical dividing cell of an animal, the centrioles change their function during the cell cycle. Centrioles form a cilium during G1, and a centrosome during the S, G2, and M phases of the cell. Unlike in sperm cells where the cilium acts a cell motor, in a typical dividing cell, the cilium functions as the cell antenna receiving external signals and transducing them into cellular signals. The centrosome functions as a major organizer of the microtubule cytoskeleton. Thus, the centrioles oscillate between the two states as cells repeatedly divide throughout animal development and adult life (Figure 4). 

A typical centrosome is made of an older centriole (the mother centriole), a younger centriole (the daughter centriole), and pericentriolar material (PCM). The centrioles are barrel-shaped structures that contain a wall made of nine triplet microtubules. The daughter centriole has a cartwheel-like structure in its proximal lumen that is not present in the mother centriole. The cartwheel is a scaffold structure that is essential for the formation of the centriole. The mother centriole has appendages around the distal tip of the centriole wall that are not present in the daughter centriole. The appendages anchor the centriole to the cell membrane, which is an essential step in forming a cilium (see below). 

The mother centriole consists of small PCM and a few astral microtubules during cilium formation. To form the cilium, the mother centriole anchors to the cell membrane via its appendages. Then, the mother centriole’s microtubule-growing end extends and forms the cilium skeleton (the axoneme). Thus, the mother centriole microtubules act as the origin that gives rise to the cilium. The mother centriole acts as the base of cilium, which is also known as basal body.

In its alternative cellular role, the mother centriole forms a centrosome by recruiting the PCM. The PCM appears as an amorphous material. However, recent studies demonstrate that the PCM consists of multiple radial layers of specific proteins around the centriole [16]. The PCM contains the protein complexes that form astral microtubules that emanate from the centrosome, reaching to the cell membrane across the cytoplasm. PCM proteins are recruited to the centriole via proteins that anchor them to the centriole lumen [17,18]. Despite many efforts, the necessity of centriolar microtubules for PCM recruitment has not been demonstrated, suggesting that other proteins within the centriole are mediating this process. Thus, centriolar proteins hold the PCM together and the PCM carries out the major function of the centrosome. 

To ensure that cells have a precise number of centrioles, new centrioles form by a process referred to as centriole duplication. In this process, the pre-existing centriole serves as a platform to make sure that a single centriole nucleates once in a cell cycle. However, the pre-existing centriole does not determine the structure of the centriole; instead, the new centriole structure is determined by the cell type that assembles it [19,20]. Since the pre-existing centriole is not a template for the new centriole, a pre-existing centriole and the new centriole can have different structures. Despite major progress in identifying the proteins that initiate centriole formation, it is not yet known how the pre-existing centriole restricts the number of new centrioles to one. The cell type determines the new centriole structure, presumably via the proteins that it expresses and their regulation, but the mechanism is unknown.

Altogether, the centriole microtubules are essential for cilium formation, but not for centrosome formation or centriole nucleation. Therefore, the distal centriole of the spermatozoon that has already formed the cilium and is not going to build a new cilium in the zygote does not need the centriole microtubules. However, the spermatozoon’s two centrioles are necessary to form centrosomes and nucleate new centrioles in the zygote. It is expected that the two sperm centrioles, even if they have atypical composition, would have the minimal protein machinery that enables these two activities.

## 3. Spermatozoa Have Specialized Centrioles

As described earlier, the strong competition among spermatozoa and the resulting rapid evolution of sperm practically ensured that sperm are always optimized for its unique task. Therefore, every structure or protein that is not necessary might be eliminated so that all of the resources are dedicated to the sperm’s highly specific function. Therefore, we can expect that spermatozoon centrioles would be simplified to the minimums that are essential for reaching the oocyte and initiating proper zygotic cell function.

The centrioles have essential functions during spermatozoa formation, spermatozoa movement, and post-fertilization. However, these functions do not necessarily require all of the components of a typical centriole. The unique features of sperm centrioles are:

- Unlike early sperm cells, the mature spermatozoon does not need an aster of microtubules. Therefore, the spermatozoon centrioles do not need to maintain a PCM.
- A typical centriole is necessary to form the sperm flagellum, but not to maintain it once it is formed. Since spermatozoon has a flagellum, the typical centriole is dispensable at that stage.
- In primitive sperm, the mature spermatozoon centriole anchors the tail to the head, which is an important function. However, this anchoring function may not be required in mammals that have specialized sperm neck structures (the striated column and capitulum) that evolved to be the anchor (Figure 1c) [21]. Therefore, the anchoring function of the typical centriole is dispensable in mammalian spermatozoa.
- Finally, the zygote and the early embryo do not need cilia, and therefore, the spermatozoon is not required to deliver a centriole that is capable of forming a cilium [22,23]. Since the ooplasm contains all of the proteins that are necessary to form centrioles and a centrosome, the sperm needs only to bring a minimal centriole that can serve as a nucleation site to recruit PCM and centriole duplication machinery.

Such minimal centrioles were recently identified in the spermatozoon of insects and non-rodent mammals (Figure 3) [9,10,11,12]. These centrioles are smaller, have reduced protein composition, and have either no microtubules or have microtubules that lack nine-fold symmetry. These atypical centrioles can recruit PCM to form a centrosome and recruit the centriole duplication machinery to form new centrioles, but would not be able to form a cilium. Thus, it is possible that the typical centriole is optimized by evolution to perform regular cell functions, yet more rudimentary versions of the centriole are optimized for spermatozoan and zygotic functions. Since centriole numbers are tightly controlled, it makes sense that the zygote would need two centrioles provided by the spermatozoan, whether they are typical or rudimentary (atypical), for the embryo’s future cells. Therefore, it is surprising that the spermatozoa of rodent mammals are thought to have no centrioles. Indeed, no centriolar-like structures or centrosomal proteins have been found to date in the sperm neck of mice [24]. This observation may reflect that either rodents have very atypical centrioles, or, as many suggest, do not have centrioles at all and that the embryo centrioles form de novo [25,26,27]. Understanding the evolutionary changes in sperm centrioles will enable us to define the minimal components for different centriole functions and the complementing requirements from the oocyte. 

## 4. Spermatids and Spermatozoa Have Two Centrioles: The Proximal Centriole and the Distal Centriole

**Figure 5:**
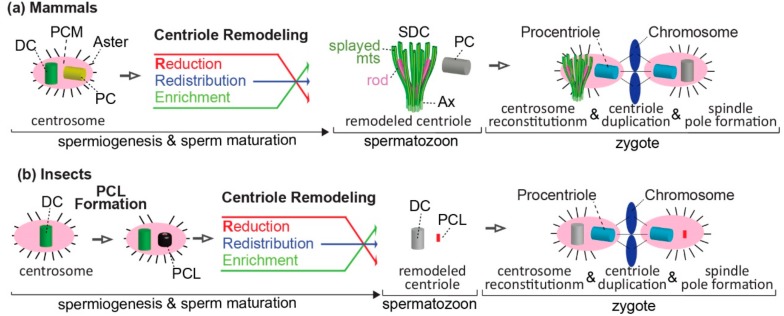
Mechanisms of atypical centriole formation in mammals and insects. (**a**) Model of centriole remodeling that transforms the typical centriole to an atypical centriole, and the role of sperm centrioles in the zygotes of non-rodent mammals. The early spermatid centrosome is made of two typical centrioles (PC and DC) and pericentriolar material (PCM) that form an aster. During spermiogenesis, centrosomal proteins are redistributed, and their amount is altered (reduced or enriched). Consequently, the DC is remodeled and forms an atypical centriole: the spermatozoon distal centriole (SDC). The SDC is made of rods and splayed microtubules (mts), which are attached to the core of the flagellum (axoneme, Ax) in the mature spermatozoon. The PC remains structurally typical, but it has an atypical protein composition. After fertilization, both the typical and atypical centrioles recruit maternal PCM and reconstitute the zygote centrosome. Later, these typical and atypical centrioles separate and each form a centrosome, an aster, a new centriole (procentriole), and a spindle pole at distinct stages of zygote development. (**b**) Model of atypical centriole formation, followed by centriole remodeling, and the role of sperm centrioles in the zygote of insect. The early spermatid centrosome consists of a single centriole that becomes distal centriole (DC). The DC is surrounded by PCM and forms an aster. During early spermiogenesis, an atypical centriole forms: the PCL. During mid and late spermiogenesis, centrosomal proteins are redistributed, and their amount is altered (reduced or enriched). Consequently, the DC protein composition is remodeled, but it maintains a centriole wall made of microtubules, which are attached to the core of the flagellum (axoneme, Ax) in the mature spermatozoon. The PCL is modified structurally and compositionally. After fertilization, both the DC and PCL recruit maternal PCM and reconstitute the zygote centrosome, which later forms an aster, a new centriole (procentriole), and a spindle pole at distinct stages of zygote development.

A dramatic cellular transformation forms the spermatozoon from a round spermatid (Figure 1). The early spermatid is similar to an undifferentiated dividing cell, in that in most animals it has two typical centrioles, with the exception of insects, where one centriole forms as an atypical centriole (Figure 2c,d and Figure 5 see below). As in somatic dividing cells, the mother centriole attaches to the early spermatid’s cell membrane and forms the cilium. As the spermatid differentiates, it develops a set of unique features that are not found in typical cells. After cilium formation, the mother centriole and daughter centriole with the cilium migrate into the cell interior and attach to the nucleus. This migration positions the daughter centriole near the nucleus; thus, it was named the proximal centriole. The mother centriole is situated farther from the nucleus, and therefore it was named the distal centriole. At this early stage of sperm formation, the centrioles start to be modified both in their structure and composition in a process that is referred to as centrosome reduction and centriole remodeling. Also, the sperm cilium formed by the centriole is uniquely modified in the sperm cell; however, these changes are beyond the scope of this review (cilium modifications were recently reviewed in Avidor-Reiss and Leroux [13]).

Unlike human and other mammals where the early spermatids have two typical centrioles, the insect’s early spermatids have only one typical centriole (Figure 2c,d and Figure 5b). This typical centriole is homologous to the distal centriole as it forms the cilium. Recently, an atypical centriole was found in the insect early spermatid, suggesting that counter to the prevailing dogma, the early spermatids of insects also have two centrioles (Figure 3a–c). This atypical centriole is thought to be the homolog of the proximal centriole, since both the proximal centriole in mammals and the atypical centriole represent the younger centriole of the cell. Therefore, it was named the proximal centriole-like structure (PCL) [11]. As in other animals at later spermatid stages, both the insect distal centriole and PCL are modified in their structure and composition by the process of centrosome reduction and centriole remodeling [15,28]. 

Altogether, animal early spermatids are initially similar to undifferentiated cells with a mother centriole (the distal centriole, or DC), a daughter centriole (the proximal centriole, or PC/PCL), and a cilium attached to the mother centriole. Both of these centrioles are later modified by centrosome reduction and the centriole remodeling process. However, two centrioles remain in the spermatozoon, and contribute to the zygote during fertilization (see below) (Figure 5).

## 5. The Diverse Types of Distal Centrioles

In all mammals, the distal centrioles were thought to degenerate and become nonfunctional as a result of centrosome reduction [29,30,31]. However, a recent detailed study found that the distal centriole is dramatically remodeled in structure and composition, yet it remains functional (Figure 3d–f) [9]. The structure of the remodeled distal centriole consists of splayed microtubules that are flanked by bars made of centriole luminal proteins at the base of the axoneme (Figure 2b), with only a subset of the centriolar proteins typically found in a centriole. Interestingly, the distal centrioles of rodent mammals appear to be missing centrin [24], which is one of the few proteins that label the remodeled distal centrioles, suggesting that rodent distal centrioles are even more modified than the distal centrioles in other mammals. 

In some insect species, such as *Drosophila melanogaster*, the base of the axoneme has the classic configuration of nine triplets of microtubules in the spermatozoa [15] (Figure 2b). In some other species, such as *Tribolium castaneum*, instead of nine triplet microtubules, the base of the axoneme has nine doublet microtubules. Doublet microtubules are expected in an axoneme, but not in a centriole. This difference caused some investigators to conclude that the *Tribolium* axoneme has lost the centriole at its base. However, the end of the axoneme base in *Tribolium* lacks the central pair of microtubules that are necessary for motile axoneme function [10,32]. Since centrioles do not normally have central microtubules, it was later proposed that the base of the axoneme of *Tribolium* is not an axoneme, but rather a centriole. This hypothesis was confirmed when it was shown that the base of the axoneme contains the centriole specific protein Ana1 [10].

In other insect species, such as the spur-throated grasshopper *Melanoplus*, the base of the axoneme of the spermatozoa has nine doublet microtubules, which are accompanied by two central microtubules [33]. Originally, the presence of the central microtubules led to the conclusion that the spur-throated grasshopper has no centrioles because central microtubules are a characteristic of the axoneme, not a centriole [33]. However, we argue that this may represent that the central microtubules migrated or grow into the distal centriole; as in many amniotes, the central microtubules have been shown to enter the distal centriole during spermatogenesis and are present in the mature spermatozoa [34,35,36,37] (see below). 

In many insect species with atypical distal centrioles in the spermatozoon, the primary spermatocyte has centrioles that have the classic configuration of nine triplets of microtubules. Since each of the two centrioles of the primary spermatocyte becomes the distal centriole of the spermatozoon, the distal centriole must have been modified (see below centrosome reduction or centrosome remodeling).

In non-mammals and non-insects, the distal centriole is thought to have a typical structure (Figure 1 and Figure 2a). However, in amniotes, such as snakes and turtles, the central pair of microtubules enter the distal centriole that has nine triplets of microtubules, which is a property that is atypical of a centriole [35,37]. Similarly, in mammals, the central pair enters the distal centriole before it degenerates [36]. This suggests that the changes in the distal centriole started early in tetrapod evolution. It would be interesting to study the composition of the distal centriole in various tetrapods and amniotes to test whether its centriole composition started changing before or during the remodeling of the distal centriole structures.

Here, our discussion focuses on the cross-section characteristics of the distal centriole; however, centrioles also deviate in length. While most animal cells have centrioles that are 400–600 nm long, the distal centriole can be much longer. The *Drosophila melanogaster* distal centriole is 1800 nm long [38]. Similarly, the ostrich (*Struthio camelus*) distal centriole is 3000 nm long [39]. The mechanism that forms these long centrioles are described elsewhere [40]. 

Altogether, many animals have distal centrioles with typical structure, but evolutionary specialization can promote considerable morphological and molecular variability. Recent accumulating data suggest that despite the dramatic modifications, the distal centriole still maintains a set of necessary functional properties. Differences are present between major groups such as mammals and insects, and also between more closely related groups such as subgroups of mammals, suggesting that the distal centrioles evolve rapidly and continuously.

## 6. The Diverse Types of Proximal Centrioles

To be typical, a centriole needs to have both structure and composition that are typical. Most animals, including non-rodent mammals, have a proximal centriole with a typical structure that is made of nine triplets of microtubules in the spermatozoon. However, this typical structure centriole may have atypical composition. In human, some of the proteins such as SAS-6, Centrobin, and CEP295 are all missing in the proximal centriole, which is normally present in a typical centriole [9]. In rodent mammals, the proximal centriole is thought to be degenerated [24]. In light of the presented discoveries of extremely diverse atypical centrioles (also see below), the apparent absence of centrioles in rodent mammals may need to be revisited.

For many decades, insect sperm was thought to lack a proximal centriole in both spermatid and spermatozoon [33,41]. However, recent studies of centriole localization proteins suggest that an atypical proximal centriole (PCL) is present in the spermatid and spermatozoon of *Drosophila melanogaster* [11]. Recent detailed electron microscopy studies of two additional insect species identified such a PCL with some structural differences [10,12,42].

The first PCL was identified in *Drosophila melanogaster* (Figure 2b,d). The Drosophila PCL lacks microtubules [11,15,42]; instead, the PCL has a novel structure consisting of a ~100-nm wide electron dense wall with a ~20-nm wide central tubule and translucent interstitial material between them [15]. Within the lumen of the PCL, the central tubule appears to have spokes. Altogether, Drosophila PCL resembles an early procentriole intermediate (after the cartwheel is formed but before the microtubules are formed, see below). 

The second PCL type was identified in the Tenebrionidae beetle, *Tribolium castaneum* (Figure 2b) [10]. As in Drosophila PCL, the Tribolium PCL lacks microtubules and has a ~100-nm wide electron dense wall. However, unlike the Drosophila PCL, the Tribolium PCL core is electron-dense, and it is not clear if it has the central tubule.

The third PCL type was identified in the coccinellid beetle, *Adalia decempunctata* (Figure 2b) [12]. The lumen of the Adalia PCL is similar to that of the *Drosophila* PCL in that it also has a ~20-nm wide central tubule surrounded by a translucent interstitial material. However, the central tubule of *Adalia* PCL has more distinguishable spokes than that of the *Drosophila* PCL. Most significantly, unlike the *Drosophila* and *Tribolium* PCL, the *Adalia* PCL has nine singlet microtubules in its electron-dense wall. In this regard, the *Adalia* PCL is more similar to procentriole. However, the typical procentriole has nine triplet microtubules.

The PCL, which is small to begin with, is further modified in the spermatid to an even smaller entity, such that it is hard to observe by electron microscopy in the spermatozoon. Therefore, some propose that the PCL degenerates in the spermatozoon [12,42]. However, a remodeled PCL can be detected by labeling the PCL specific protein POC1B in flies [15]. Similarly, similar to the PCL, the fly protein composition of the DC is also remodeled [28]. Moreover, several independent lines of investigation demonstrate that the fly sperm provides two centrioles to the zygote: one is the typical centriole (i.e., the DC), and the other is the PCL [43]. Therefore, flies provide both a remodeled distal centriole and remodeled PCL to the embryo. It is likely that other insects also provide two sperm centrioles to the embryo, but this needs to be tested.

In summary, as observed with the distal centriole, the proximal centriole can have a range of structures, and proximal centriole differences are present both between major groups and subgroups of animals. However, while all of the known distal centrioles form initially as typical centrioles and later become atypical by remodeling, the insect proximal centriole is already formed as an atypical centriole (PCL), and later it is modified further by centriole remodeling. Also, the centriole’s microtubules appear to be affected in various ways, including their absence or splaying. Since the centriole microtubules are essential for cilium formation, an atypical proximal centriole that lacks microtubules cannot form a primary cilium. Also, since the axoneme is attached to the distal centriole microtubules, the distal centriole cannot form a primary cilium. However, because the centriole microtubules are dispensable for centrosome formation, an atypical proximal or distal centriole that lacks microtubules or has attached axonemal microtubules can recruit the pericentriolar material (PCM) and form a centrosome. Since the early embryo does not have a cilium, but does have a centrosome, the sperm atypical and typical centrioles can perform their necessary function post-fertilization (Figure 5). 

## 7. Atypical Sperm Centrioles Are Formed by Both Common and Distinct Mechanisms, Suggesting a Combination of Common Origin and Convergent Evolution

Typical centrioles are surrounded by PCM that anchors and nucleates the astral microtubules. In all of the groups that were investigated, the spermatozoa centrioles lose the astral microtubules, and most if not all of the PCM during spermiogenesis as part of the well-characterized process of PCM/centrosome reduction [29,30]. Since all of the groups examined share these losses, this trait might have been present in the original ancestor of all animals, and may be of common origin. 

Typical centrioles have a wall made of microtubules with nine-fold symmetry. Atypical centrioles deviating from this structure are present in the sperm of both insects and mammals. These atypical structures share both similarities and differences between the two groups in the biological processes that generate them. The two animal groups are similar in that the centriole remodeling takes place after the centriole and cilium formation. However, the two animal groups differ in some details of the centriole remodeling. 

First, in mammals, the most modified centriole is the distal centriole, and in insects, the most modified centriole is the proximal centriole. This may reflect a difference in a yet unknown role of the proximal and distal centriole in the mature sperm of insects and mammals. Nonetheless, this remodeling results in mammals and insects getting one typical centriole and one atypical centriole, but which centriole in the sperm gets modified is different from group to group. Since many animal groups have two typical centrioles, the difference in the identity of the atypical centriole argues that evolving an atypical centriole may be due to convergent evolution. 

Second, in mammals, the atypical centriole is formed originally as a typical centriole, which later is remodeled, while in insects, the atypical centriole forms as an atypical structure, which later is remodeled to become more atypical (Figure 2c,d). This difference in the original structure raises an interesting question on the evolutionary steps that led to the formation of the atypical insect centriole. Since flies and flower beetles have originally a more basic atypical centriole (PCL with no microtubules) than the atypical centriole of *Adalia* (PCL with singlet microtubules) (Figure 2b), it is possible that forming the atypical centriole rather than modifying a typical centriole is an evolutionary advancement of the remodeling process. 

Furthermore, there is evidence from the phylogeny of both mammals and insects that the centriole remodeling process evolved independently. Mammals are a subgroup of amniotes, and non-mammalian amniotes, such as snakes and turtles, have a distal centriole in the spermatozoon that has nine triplets of microtubules [35,37]. Therefore, it was suggested that the mammalian atypical centriole is a derived (recently evolved) trait, and is not ancestral to all amniotes [37]. Similarly, insects are a subgroup of arthropods, and other arthropods, such as scorpions and spiders, have a typical proximal centriole that has nine triplets of microtubules [44,45]. Consequently, the atypical insect centriole also appears to be a derived trait that is not ancestral to all arthropods.

Altogether, one hypothetical model is that centriole remodeling evolved in several steps. The first evolutionary step was to lose astral microtubules and the PCM. This happened in the animal ancestor.

The second evolutionary step happened independently in amniotes/mammals and insects. In amniotes, the second evolutionary step is the entrance of central microtubules that are normally restricted to the axoneme into the distal centriole. Later, in the mammal ancestor, the third evolutionary step was the splaying of the nine triplets of the distal centriole and converting them to nine doublets. In insects, the second evolutionary step has two components: (1) forming a partially modified centriole (*Adalia* procentriole formation in the spermatid that has single microtubules) that is followed by (2) a remodeling process to reach the final atypical state. Whether these two appeared simultaneously or in a step manner requires further study of more insect groups. 

The third evolutionary step is to further simplify the formation of the atypical centriole as it is observed in flies and flower beetles to a PCL with no microtubules.

It is also interesting that in insects, the final spermatozoan PCL is formed in two steps; we do not know of any cases in which an atypical centriole becomes atypical without the second remodeling step. This may suggest that the PCL must keep some of its structure in the early spermatid. What can be the function of the PCL in early spermatids? During spermiogenesis, the nucleus is shaped by a transient microtubule-based structure, the manchette [46,47]. It was proposed that the proximal centriole and its centriolar adjunct functions as the site of nucleating or anchoring the manchette microtubules [36]. Therefore, one possibility is that the initial PCL structure is essential for manchette formation or function. 

Since the atypical centrioles can be produced through both lineage’s shared processes (PCM/centrosome reduction) and linage-specific processes (centriole remodeling), it is possible that both a shared origin and a convergent evolution mechanism are in play. Addressing this question requires identifying the function of centriole remodeling and the evolutionary advantage that it provides. Also, to determine this shared origin, it would be valuable to define the underlying molecular modifications and mechanisms during centriole remodeling. 

## 8. What Is the Evolutionary Mechanism Underling Sperm Centrioles’ Diversity?

The diversity in the sperm centriole structure can be driven by the selection of advantageous adaptations, which make the sperm better fit, or by drift, which is a consequence of random sperm changes. Both selection and drift can be driven by a mechanism of relaxed constraint. An example of advantageous adaptations is that the mammalian atypical distal centriole has a bilateral symmetry similar to the rest of the sperm neck and tail (Figure 1c), potentially making the sperm movement more competitive than with a typical distal centriole. An example of a relaxed constraint is that cilia are not needed in early embryo development, and therefore, sperm centrioles can lose any property that is needed to form cilium (e.g., microtubules), as long as they maintain other abilities that are needed in the embryo such as new centriole nucleation, centrosome formation, and aster formation.

The selection of advantageous adaptations can be due to gaining new centriole traits that benefit the sperm’s ability to fertilize the egg. A possible mechanism for such advantageous adaptations of a centriole trait may include the remodeling of the mammalian distal centriole to improve sperm beat. Unlike other spermatozoa that beat their tails in a circular motion, mammalian sperm can beat more linearly from left to right [48]. Also, the primitive sperm has a sperm tail that has radial symmetry mirroring the radial (nine-fold) symmetry of the centriole, axoneme, and the radial movement (Figure 1b). In contrast, the mammalian sperm tail has accessory structures around the centriole and axoneme that have a bilateral symmetry that mirrors the tail linear movement (Figure 1c). Since the remodeling of the distal centriole changes the distal centriole symmetry from nine folds to bilateral, allowing the centriole with the symmetry of the accessory structures, it is possible that this change is advantageous for the sperm beat pattern.

Alternatively, advantageous adaptations can be due to a selection beneficial trait of a sperm function that is not related to the centriole, but happens to affect the centriole in a way that it does not outweigh the overall benefit from the trait (pleiotropy). A possible mechanism for such pleiotropy may be the function of the centrioles in shaping the sperm head. The sperm has two major types of microtubule base systems: (1) the centriole–cilium complex that forms the sperm tail, and (2) the manchette that shapes the sperm nucleus. Many genes that are essential for the centriole–cilium complex are also essential for the manchette (Lehti and Sironen, 2016 [46]). Therefore, a mutation in a gene affecting the manchette may affect the centriole and vice versa. The sperm head is one of the most variable structures of sperm cells between species/groups/subgroups, and therefore, the manchette that shapes the sperm head may be under strong selective pressure [49]. If the manchette gene mutation has a beneficial value for sperm head and simultaneously changes the centriole without a major effect on sperm function, this mutation will be selected. Indeed, a recent paper suggests that some centriole proteins have a role that extends beyond the expected role of centriole–cilium formation, and affects the formation of the sperm head [50]. In this study, a rat mutation in the centriole protein Centrobin revealed that it has an essential role in sperm head shaping. Therefore, over time, animals may accumulate many mutations that modified the centriole dramatically, because they gain an advantage in shaping the sperm head. 

The PCL may be a case of centriolar neoteny. Neoteny is an evolutionary adaptation that prolongs youthful characteristics [51]. In other words, the PCL may evolve by mutations in genes that are essential for centriole maturation, keeping the PCL from maturing and maintaining its early structural characteristics. Centriole maturation is a multistep process that takes about two cell cycles (Figure 4). The first step in centriole formation is the formation of a procentriole in the right angle to the pre-existing centriole and attached to its the base. Procentriole formation can be further divided to early, intermediate, and late stages. The early procentriole is made of a cartwheel surrounded by a dense wall, resembling the *Drosophila* PCL. The intermediate procentriole has in its wall nine singlet or doublet microtubules, resembling the *Adalia* PCL. The late procentriole has a wall made of nine triplet microtubules. Therefore, the insect atypical proximal centriole appears to represent an early-stage centriole that did not mature into a typical centriole. Over evolutionarily time, PCL can form simply by mutations that disable the maturation of the procentriole.

## 9. Concluding Remark and Future Direction

In many animals, the sperm has two typical centrioles (that sometimes are slightly modified), providing a simple explanation of how centrioles are inherited in reproduction and how the zygote generates two daughter cells. However, since the egg does not have centrioles, it is not clear how the zygote can generate two daughter cells when a spermatozoon has only one centriole. A solution to this enigma was the discovery of the atypical sperm centriole. The first sperm atypical centriole was described less than 10 years ago in the fly. Since then, atypical sperm centrioles were reported in other insects, mammals, and humans. However, in contrast to the typical centriole that demonstrates a very conserved and stereotypical structure, atypical sperm centrioles vary dramatically both between and within lineages, suggesting that they evolve rapidly and form by multiple mechanisms. How centrioles are formed at the molecular level and for what purpose is largely unknown. Similarly, it is not clear what type of evolutionary mechanisms mediate their development. Gaining insight into the molecular mechanisms that underlie atypical centriole formation and function in various animals would allow us to synthesize a hypothesis that would explain the roles and mechanisms of both the typical and the atypical centriole.
